# Supplementary material for: CDR1 Composition Can Affect Nanobody Recombinant Expression Yields
Source: Biomolecules. 2021 Sep 14;11(9):1362. doi: 10.3390/biom11091362 (PMC8465892; doi:10.3390/biom11091362)
Supplement: Supplementary file 1 [file biomolecules-11-01362-s001.zip › biomolecules-1360554-supplementary.pdf]

Supplementary Material

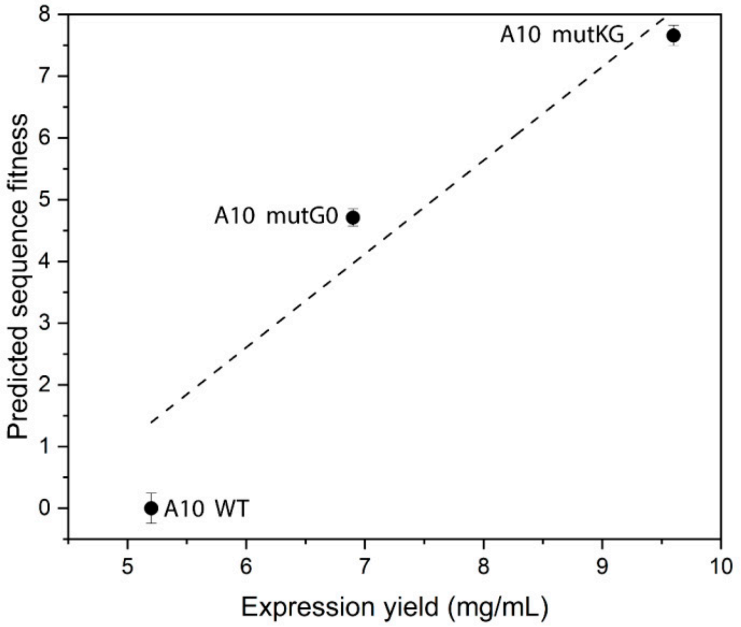

Figure S1. Linear regression of A10 variants expression yields and their predicted sequence fitness (expressed as log odds relative to A10 wt set as 0). Sequence fitness values were calculated on primary sequence with SeqDesign (https://github.com/debbiemarkslab/SeqDesign) trained on the same dataset as that used in https://doi.org/10.1038/s41467-021-22732-w.

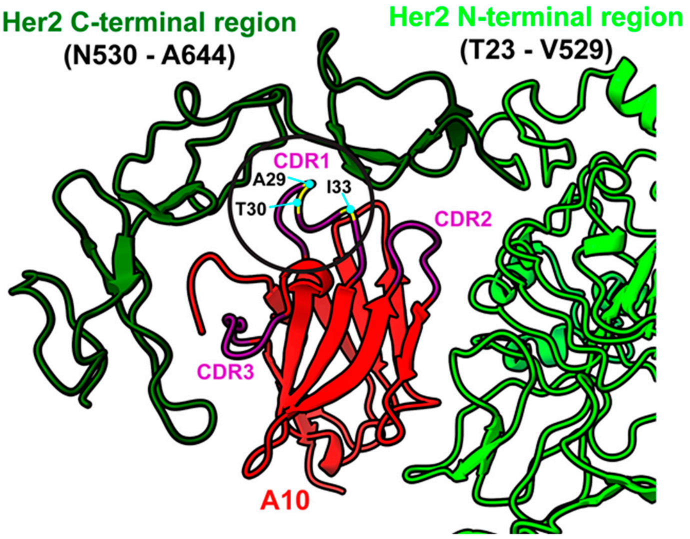

Figure S2. A10 wt/Her2 complex. The A10 wt/Her2 complex, represented in cartoon style, used as the starting structure in this study to predict the effect of glycine insertions on CDR1. This model was obtained after experiment-based docking and MD refinement [15]. CDR1 is highlighted by a black circle, and the residues predicted to determine the affinity with Her2 are depicted in yellow and labelled. The numbering of Her2 residues follows the sequence deposited in Uniprot (ID P04626).

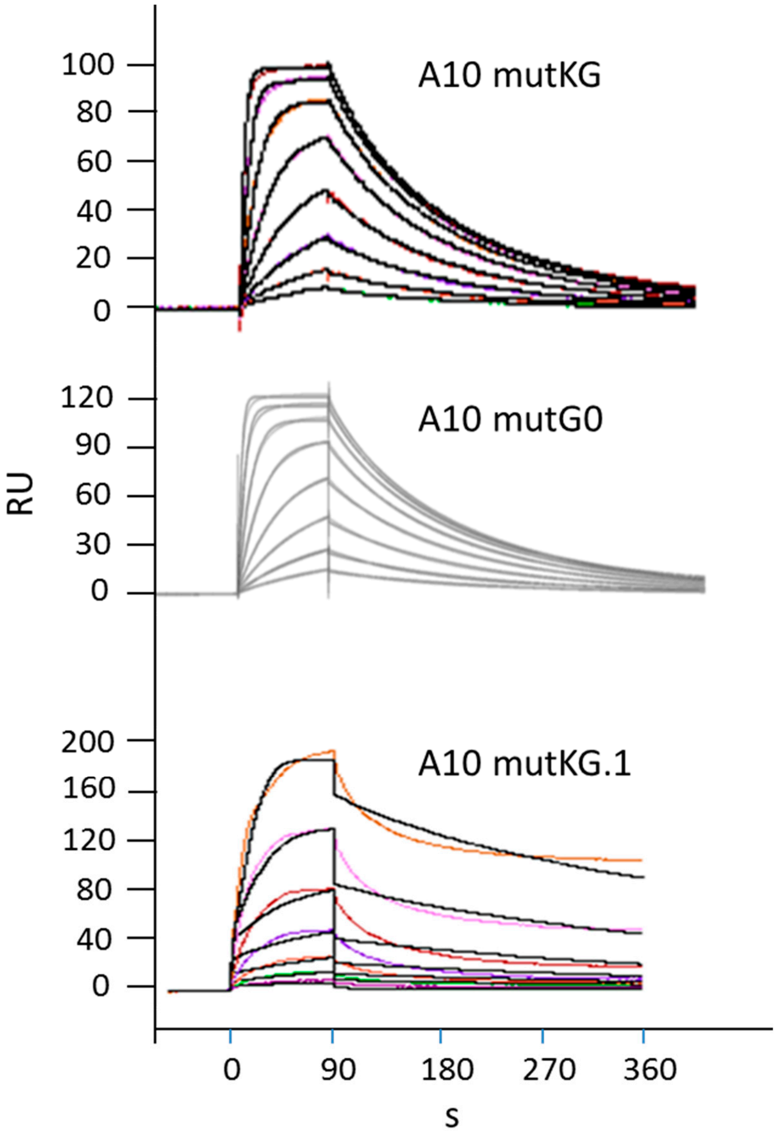

Figure S3. SPR analysis. The curves corresponding to the binding kinetics of the A10 mutants KG, G0 and KG.1 are reported

Table S1. SeqDesign assessment of CDR1 mutant fitness.

Insertion Mutants	Logarithmic Differences of Relative Probability
AgTSNISN (mutKG)	7.66 ± 0.16
ATgSNISN	5.88 ± 0.14
ATSgNISN (mutG0)	4.71 ± 0.14
ATSNgISN	5.76 ± 0.13
ATSNIgSN	5.59 ± 0.12
ATSNISgN	.98 ± 0.13

The effect of the insertion of a single glycine at different positions of the A10wt CDR1 (ATSNISN) was analyzed. Values are logarithmic differences of relative probability, higher they are and better are the fitness chances of the variant

Table S2. List of non-covalent Her2/A10 variants interactions.

Her2 Residue	Region	A10 Residue	Region	Interaction Type	% Simulation Frames		
					WT	mutKG	mutG0
Y554	Sidechain	A29	Backbone and sidechain	Hydrophobic	85	30	-
Y554	Backbone and sidechain	I33 (or I34)	Sidechain	Hydrophobic	82	56	62
C563-C576	Sidechain	A29	Backbone and sidechain	Van der Vaals	81	64	81
N555	Sidechain	I33 (or I34)	Sidechain	Van der Vaals	80	87	77
L561	Sidechain	I33 (or I34)	Sidechain	Hydrophobic	64	74	79
P594	Sidechain	T30 (or T31)	Sidechain	Van der Vaals	52	35	-
L561	Sidechain	A29	Backbone and Sidechain	Hydrophobic	51	66	66
Y554	Sidechain	T30 or (T31)	Backbone and Sidechain	Hydrogen bond	42	-	31
F595	Sidechain	T30 (or T31)	Sidechain	Hydrophobic	21	87	64
P594	Sidechain	T30 (or T31)	Sidechain	Hydrogen bond	29	-	30
Y554	Sidechain	G30	Backbone	Van der Vaals	-	77	-
Y554	Sidechain	A29	Backbone	Hydrophobic	-	-	76
F577	Backbone	G30	Backbone	Hydrogen bond	-	12	-

Interactions lasting over more than 10% of MD simulations frames of A10 variants/Her2 complex are reported. Interactions lasting for more than 50% of the simulation frames are highlighted in yellow.
